# Efficacy and Safety of Ketamine-assisted Electroconvulsive Therapy in Major Depressive Episode: A Systematic Review and Network Meta-Analysis

**DOI:** 10.21203/rs.3.rs-3182771/v1

**Published:** 2023-08-07

**Authors:** Taeho Greg Rhee, Sung Ryul Shim, Jonah Popp, Thomas Trikalinos, Robert Rosenheck, Charles Kellner, Stephen Seiner, Randall Espinoza, Brent Forester, Roger McIntyre

**Affiliations:** Yale School of Medicine; Yale Medical School; Departments of Neurology, Psychiatry and Biobehavioral Sciences, University of California

**Keywords:** ketamine, electroconvulsive therapy, depression

## Abstract

**Objective:**

To meta-analyze clinical efficacy and safety of ketamine compared with other anesthetic agents in the course of electroconvulsive therapy (ECT) in major depressive episode (MDE).

**Methods:**

PubMed/MEDLINE, Cochrane Library, Embase, GoogleScholar, and US and European trial registries were searched from inception through May 23, 2023, with no language limits. We included RCTs with (1) a diagnosis of MDE; (2) ECT intervention with ketamine and/or other anesthetic agents; and (3) measures included: depressive symptoms, cognitive performance, remission or response rates, and serious adverse events. Network meta-analysis (NMA) was performed to compare ketamine and 7 other anesthetic agents. Hedges’ *g* standardized mean differences (SMDs) were used for continuous measures, and relative risks (RRs) were used for other binary outcomes using random-effects models.

**Results:**

Twenty-two studies were included in the systematic review. A total of 2,322 patients from 17 RCTs were included in the NMA. The overall pooled SMD of ketamine, as compared with a propofol reference group, was −2.21 (95% confidence interval [CI], −3.79 to −0.64) in depressive symptoms, indicating that ketamine had better antidepressant efficacy than propofol. In a sensitivity analysis, however, ketamine-treated patients had a worse outcome in cognitive performance than propofol-treated patients (SMD, −0.18; 95% CI, −0.28 to −0.09). No other statistically significant differences were found.

**Conclusions:**

Ketamine-assisted ECT is tolerable and may be efficacious in improving depressive symptoms, but a relative adverse impact on cognition may be an important clinical consideration. Anesthetic agents should be considered based on patient profiles and/or preferences to improve effectiveness and safety of ECT use.

## Introduction

Major depressive disorder (MDD) is one of the most common and disabling mental health conditions, affecting about 15.7 million adults aged ≥18 years in the US.^1–3^ Unfortunately, more than 30% of individuals with MDD do not achieve remission after two or more trials of antidepressants.^4,5^ Such treatment-resistant depression (TRD) is associated with premature mortality including suicide,^6–8^ reduced functioning and poorer quality of life.^9,10^

Electroconvulsive therapy (ECT) is highly effective in treating MDD, with response rates around 70% and remission rates around 50% even in those with TRD.^11,12^ Yet, ECT is often considered only after multiple prior treatment failures due in part to provider barriers (e.g., availability of well-trained ECT practitioners and physical space) and patient barriers (e.g., stigma or public attitudes).^13^ Furthermore, ECT has been criticized for adverse cognitive effects even though the risk for these side effects has been reduced by recent procedural changes (e.g., right unilateral ECT with ultra-brief pulse width).^14^

Anesthetic techniques have become increasingly refined to improve patient tolerability and safety of ECT.^15^ To date, there are multiple anesthetic agents utilized in the delivery of ECT such as: methohexital, thiopental, propofol, diazepam, ketamine, and etomidate. Of these agents, intravenous ketamine, a glutamate *N*-methyl-D-aspartate (NMDA) receptor antagonist, has gained attention since early 2000s for its rapid-acting antidepressant properties.^16,17^ Several studies also have reported that, additionally, ketamine has rapid effects at reducing suicidal ideation in patients with mood disorders.^18–21^

Janssen Pharmaceuticals has recently developed and investigated the safety and efficacy of an intranasal (IN) formulation of the S-enantiomer of ketamine (i.e., IN esketamine). Intranasal delivery was chosen as preferable to IV administration to increase patient acceptance and accessibility in a broader range of psychiatric care settings. In March 2019, the US Food and Drug Administration (FDA) approved IN esketamine (Spravato^®^) as a therapy for TRD in the form of a nasal spray.^22^ In August 2020, additional data led to a FDA approval for use of Spravato^®^ in MDD with suicidal ideation.^23,24^

While ketamine was initially introduced as an anesthetic alternative to phencyclidine (PCP) in the 1960s,^17,25^ it is unclear whether use of ketamine as the anesthetic in ECT (i.e., ketamine-assisted ECT) results in better efficacy and safety outcomes when compared to other anesthetic agents. Several studies highlighted the importance of resolving this issue,^26,27^ but no study has yet quantified the overall treatment effect sizes of efficacy and safety outcomes between ketamine-assisted ECT and ECT inducted by other anesthetic agents, using a network meta-analysis, which allows multiple treatment comparisons. This study aims to compare anesthetic agents for ECT using a systematic review and network meta-analysis of existing clinical trials.

## Methods

### Search strategy

The protocol pertaining to this study was registered on PROSPERO (CRD42023427295). A systematic search was conducted from dabatase inception to May 23, 2023. The following databases were systematically searched: PubMed/MEDLINE, the Cochrane Library, and Embase using Medical Subject Headings (MeSH) terms and text keywords. We also manually searched all relevant studies in NIH-funded clinical trial registries (https://www.clinicaltrials.gov), European (EU) clinical trial registries (https://www.clinicaltrialsregister.eu), and GoogleScholar (https://scholar.google.com/). No language restrictions were imposed. Search strategies are provided in eTable 1 in the Supplement. This study followed the preferred reporting items for systematic reviews and meta-analyses (PRISMA) reporting guidelines.^28^ Our study used publicly available data and did not include human participant research. As per 45 CFR §46.102(f), this study was not submitted for institutional review board approval and did not require informed consent procedures.

### Eligibility criteria and study selection

Inclusion criteria were established prior to article reviews and were as follows: (1) patients with a diagnosis of MDE using standardized diagnostic criteria (e.g., *Diagnostic and Statistical Manual of Mental Disorders, fifth Edition* [DSM-5] or *International Statistical Classification of Diseases and Related Health Problems, Tenth Revision* [ICD-10]); (2) ECT with intervention/comparator groups consisting of ketamine and other anesthetic agents; and (3) severity of depressive symptoms, cognitive performance, remission rates, or response rates as an efficacy outcome using validated measures (e.g., Montgomery Asberg Depression Rating Scale [MADRS] and Hamilton Depression Rating Scale [HDRS] for depressive symptoms; Montreal cognitive assessment [MOCA] and mini-mental state examination [MMSE] for global measures of cognitive performance); and (4) human-based clinical trials. We also considered safety-related events as secondary outcomes (e.g., reports of serious adverse events). Exclusion criteria were (1) non-human studies and (2) no use of validated measures for MDE or outcomes of interest.

### Study identification and data extraction

Titles and abstracts were independently screened by two reviewers (T.G.R. and S.R.S.), and articles identified as potentially relevant by at least one reviewer were retrieved and duplicates were removed. Full-text articles were independently screened by the same reviewers, and discrepancies were resolved through group discussions. Data from included articles were independently extracted by two reviewers (T.G.R. and S.R.S.) using a pilot-tested data extraction form and then corroborated, with discrepancies resolved through group discussions. Information to be extracted was established a priori and included: study characteristics (e.g., PICOTS framework), participant characteristics and subgroups, sample source and collection period, modes of ascertainment, methods of data analysis, selection of cases and controls, and quantitative data pertaining to any primary and secondary outcomes along with adjusted factors. To ensure the absence of overlapping data and to maintain the meta-integrity, data and references for each included study were carefully cross-checked.

### Assessment of risk of bias and methodological quality

The risk of bias and methodological quality were evaluated using the Cochrane Collaboration Risk-of-Bias tool version 2.^29,30^ We assessed 5 domains, including (1) randomization process, (2) deviations from the intended interventions, (3) missing outcome data, (4) measurement of the outcome, and (5) selection of the reported result. Each domain was classified as being indicative of a *high*, *low*, or *unclear* risk of bias. We used Cochrane Library’s Review Manager software, RevMan version 5.4.1,^31^ to organize extracted information on the risk of bias.

Publication bias is always a concern for evidence synthesis, and we tried to mitigate it by searching prospective trial registries. For analyses with at least 10 trials,^32^ we used funnel plots to visually explore for evidence of association between trials’ effect sizes and statistical precision.^33^ We supplemented visual assessments with statistical testing of funnel plot asymmetry using Egger’s test (i.e., a weighted linear regression of effect size versus precision) and Begg and Mazumdar’s test (i.e., rank correlation test). Evidence of associations between effect sizes and precision across studies may indicate design heterogeneity, chance or selection biases that operate cross the evidence base (e.g., publication and outcome reporting bias).

### Statistical analysis

The network meta-analysis (NMA) was performed to compare 7 different anesthetic agents and placebo used in ECT and to assess efficacy and safety in the acute phase of MDE. The NMA is advantageous as it allows multiple comparisons across different agents. In our main NMA, we compared: [1] ketamine versus [2] ketamine+propofol versus [3] propofol versus [4] methohexital versus [5] thiopental versus [6] thiopental+ketamine versus [7] midazolam versus [8] placebo (i.e., thiopental+saline). We also performed two sensitivity analyses that were limited to the most commonly used anesthetic agents: a) comparison among [1] ketamine versus [2] ketamine+propofol versus [3] propofol; and 2) comparison between [1] ketamine versus [2] methohexital. Arm-based analyses were performed to estimate the Hedges’ *g* standardized mean difference (SMD) of depressive symptoms and cognitive performance. Relative risk (RR) was used for remission and response rates and series adverse events (SAEs).

We conducted NMA analyses using ‘*netmeta’* package in R software (version 4.2.2; The R Foundation, Vienna, Austria), which applies a frequentist method based on a graph-theoretical approach in line with the electrical network theory.^34^ Using arm- and contrast-based models,^34,35^ the ‘*netmeta’* function takes within-study correlations into account by re-weighting all of each multi-arm study’s comparisons based on back-calculation of variances using the Laplacian matrix and its pseudoinverse.^35^ Because included studies were deemed to be heterogenous, we applied random-effects models.^36^

We tested the consistency of each network using split-node and design-decomposition analyses. For each comparison with direct data, the split node approach compares whether the effect from the direct data is concordant with that inferred from indirect-only data. The design-decomposition approach groups trials according to the subset of treatments they compare and assesses the concordance of findings across these groups. In addition, to assist the interpretation of treatment rankings, we estimated the probability that each treatment is among the top performing ones ( ) based on the NMA model, and calculated the surface under the cumulative ranking curve (SUCRA); a higher SUCRA value indicates a potentially better intervention.^35^

Finally, as mentioned earlier, we assessed publication bias (or small-study effects) using funnel plots;^33^ Egger’s test and Begg and Mazumdar’s test were also performed when assessing the publication bias.^33^ Unless otherwise noted, a two-sided *p*-value<0.05 was considered statistically significant.

## Results

### Characteristics of included studies

The literature search yielded 1,341 articles, of which 338 were duplicates. After screening titles and abstracts, 60 articles were eligible for full-text screening. Two independent investigators (TGR and SRS) discovered one additional study by manually searching clinical trial databases and reference lists. Details of study selection are provided in eFigure 1 in the Supplement. Overall, 22 RCTs^37–58^ were included in the systematic review (Table 1). Of these, 17 RCTs consisting of 2,322 patients met selection criteria for quantitative analyses (i.e., 17 for depressive symptoms; 6 for cognitive performance; 9 for remission; 8 for response; and 11 for SAEs). Five studies were not included in the meta-analysis due to a study design^53^ or insufficient data.^39,43,49,55^ All of the included RCTs were conducted in single sites. Seven studies (31.8%) were conducted in the US or Canada, 4 studies (18.2%) were completed in Europe, 10 studies (45.5%) were performed in Asia, and 1 study (4.5%) was completed in Australia. All studies followed RCT designs and 16 of them (72.7%) followed double-blind RCT designs. All studies recruited patients who were ECT candidates. Sample sizes for ECT with ketamine ranged from 7 to 80, and 11 studies (50.0%) also considered bipolar depression in their inclusion criteria. 21 studies (95.5%) had an ECT for 2–3 sessions a week and all studies were completed within a month. Table 1 provides details of characteristics (including frequency and duration of treatment sessions) and summaries of key findings for all included studies.

### Ketamine compared with all other anesthetic agents: A full network meta-analysis

**Figure 1:**
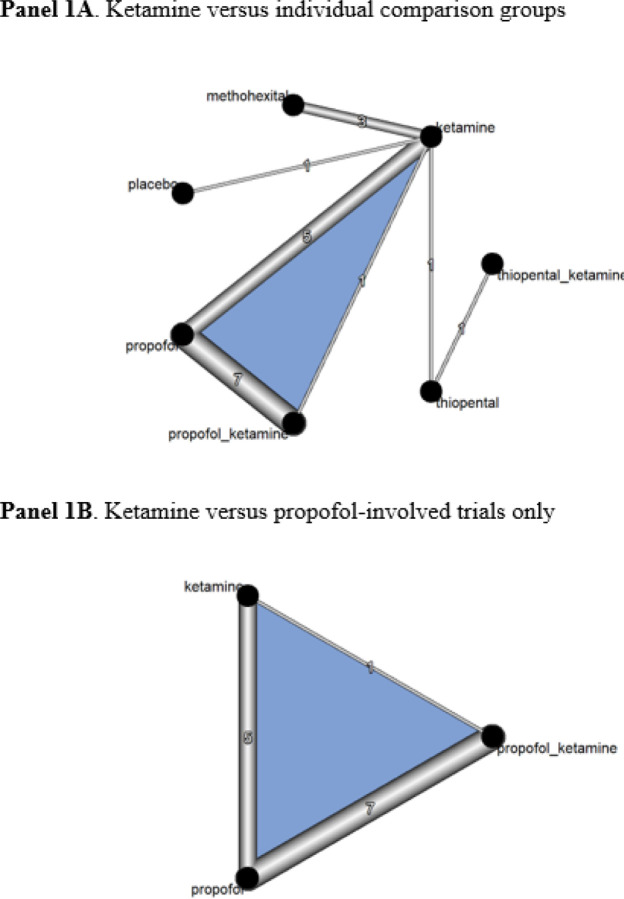
Network diagram for changes in depressive symptoms **Note:** The thickness of the lines proportional to the number of studies evaluating each direct comparison. Placebo refers to “thiopental plus saline.”

**Figure 2:**
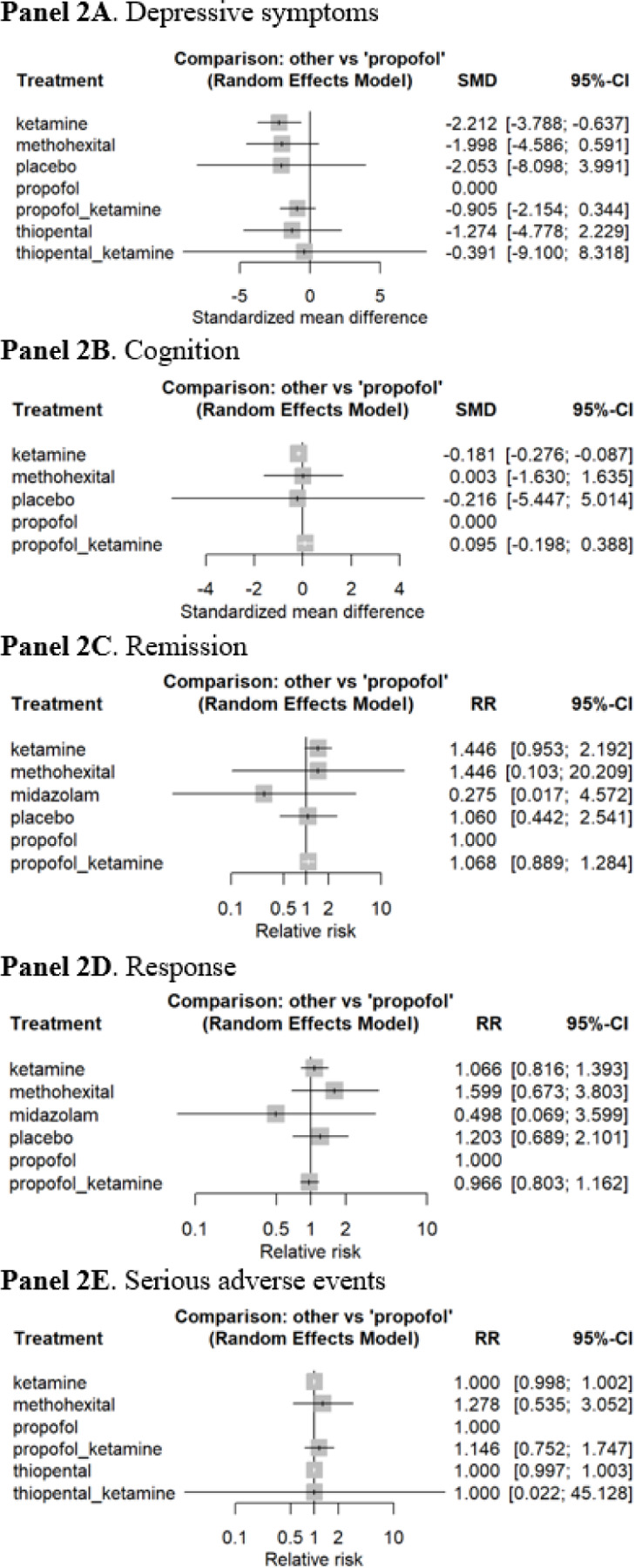
Forest plots of ketatmine versus other anaesthetic agents in the course of ECT **Note:** Placebo refers to “thiopental plus saline.” The reference group was propofol. Propofol was favored when SMD<0 for depressive symptoms or SMD>0 for cognitive outcomes. For remission and response rates, relative risk <1.0 indicates that propofol is favored. For serious adverse events, relative risk >1.0 indicates that propofol is favored.

A total of 2,322 patients from 17 direct comparison RCTs were included (i.e., 7 anesthetic agents with 19 effect sizes; Fig. 1A). In our NMA, the overall pooled SMD of ketamine, as compared with propofol as a reference group, was − 2.21 (95% confidence interval [CI], −3.79 to −0.64) in depressive symptoms, indicating that ketamine was more efficacious than propofol at reducing depressive symptoms (Fig. 2A). Ketamine was not significantly different when compared to propofol in terms of cognitive performance (Fig. 2B), remission and response rates (Figs. 2C–2D), or SAEs (Fig. 2E).

### Ketamine compared with propofol-involved agents: A sensitivity analysis

**Figure 3:**
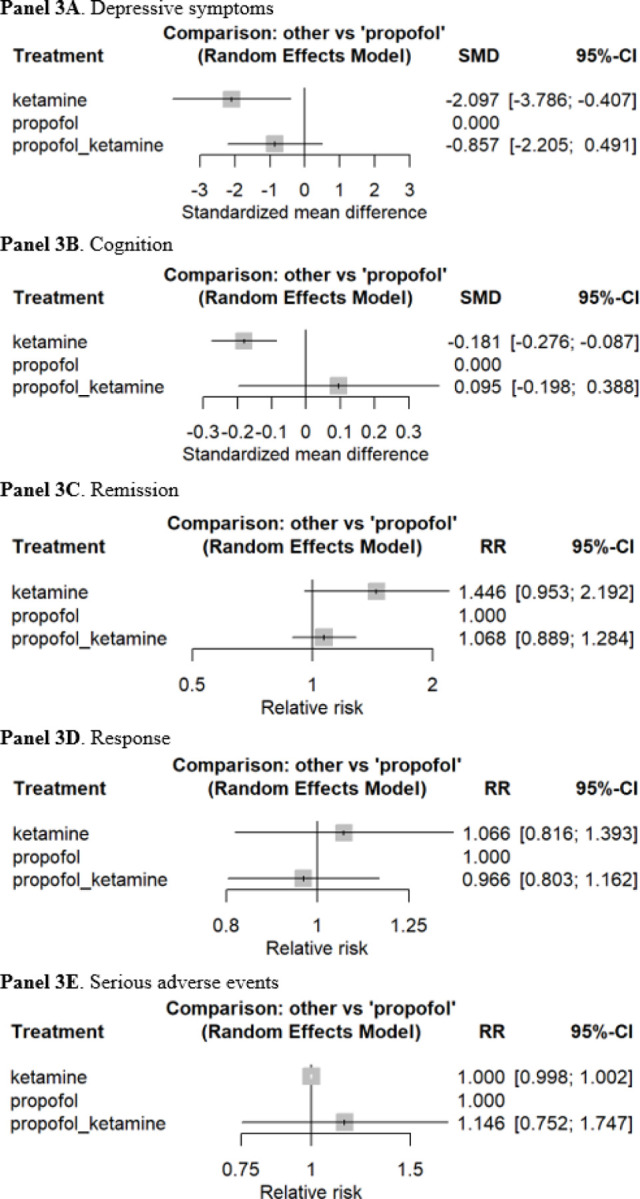
Forest plots of ketatmine versus propofol in the course of ECT **Note:** The reference group was propofol.

In our sensitivity analysis comparing [1] ketamine versus [2] ketamine + propofol versus [3] propofol, a total of 1,968 patients from 11 direct comparison RCTs were included (i.e., 3 anesthetic agents with 13 effect sizes; Fig. 1B). The overall pooled SMD of ketamine, as compared with propofol as a reference group, was − 2.10 (95% CI, −3.79 to −0.41), indicating that ketamine was better than propofol for improving depressive symptoms (Fig. 3A). However, propofol was significantly better than ketamine for cognitive performance (SMD, −0.18; 95% CI, −0.28 to −0.09) (Fig. 3B). The combination of ketamine and propofol was better than propofol alone for cognitive performance but it was not statistically significant. No statistical difference was found in terms of remission and response rates and SAEs (Figs. 3C–3E).

### Ketamine compared with methohexital: A sensitivity analysis

We also reported a pairwise meta-analysis between ketamine and methohexital (eFigure 2). We did not find any statistically significant differences in overall changes of depressive symptoms or cognitive performance. These studies did not report other outcomes.

### Inconsistency tests and Rankograms of SUCRA

**Figure 4:**
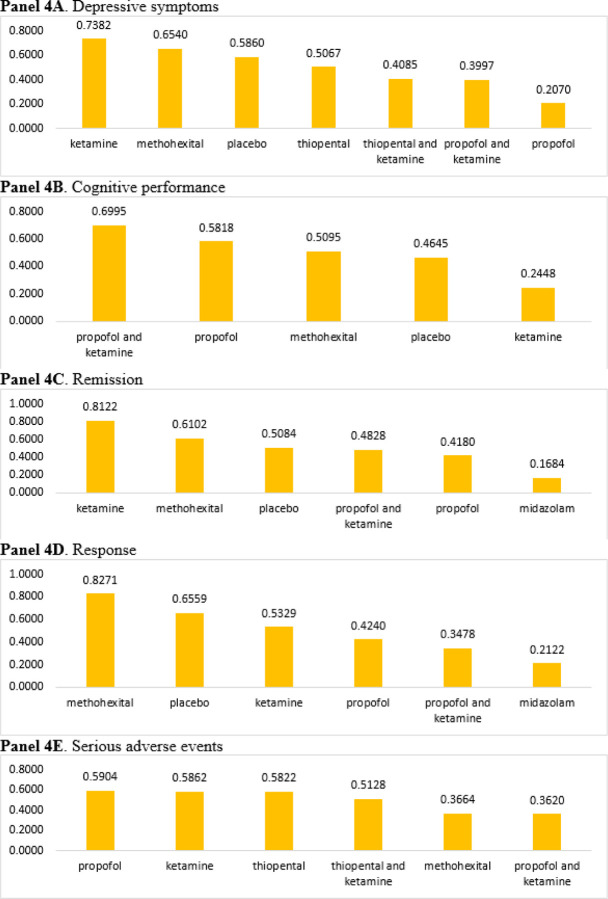
Rankograms of surface under the cumulative ranking (SUCRA) curves by each outcome measure **Note:** Graphical summary of P-scores of different interventions in the course of electroconvulsive therapy for major depressive episode. Higher and closer-to-1 P-scores indicate greater likelihood of top-rank interventions. Placebo refers to “thiopental plus saline.”

The inconsistency tests for NMA were analyzed using the node-splitting approach, and the findings (*p* > 0.05) indicate consistency across the direct and indirect comparisons of all outcomes. Figure 4 presents the SUCRA values for each anesthetic agent performance based on our full NMA model. Ketamine had the highest probability (73.8%) for depressive symptoms (Fig. 4A), indicating that ketamine may be a preferred anesthetic agent when compared to other agents. Ketamine, on the other hand, also had the poorest outcome in cognitive performance (Fig. 4B).

### Publication bias assessment

In our NMA, we did not find any evidence for potential publication bias (eFigure 3) using funnel plots and both Egger’s test (*p* > 0.05) and Begg and Mazumdar’s test (*p* > 0.05), respectively.

### Assessment of bias and methodological quality

Methodological quality of the included studies was ‘low’ to ‘high,’ and we provided our justification on eFigure 4 and 5. 9 of 22 studies (40.9%) had ‘*some concerns*’ as they may have some bias arising from the randomization process or deviations from the intended intervention. A total of 5 studies (22.7%) were deemed to have ‘*high’* risk of bias. All included studies did not have bias due to missing outcome data, resulting in ‘*low’* risk for domain 3.

## Discussion

This is the first study to use a network meta-analysis to examine the efficacy and safety of ketamine as an anesthetic agent, as compared with other anesthetic agents, in the acute phase treatment of a MDE with ECT. A network meta-analysis of 2,322 patients from 17 RCTs suggests that ketamine-assisted ECT resulted in significantly greater improvement in depressive symptoms, and the effect size was considered large.^59^ However, in our sensitivity analysis that compared ketamine with propofol or propofol plus ketamine, ketamine had an inferior outcome with respect to cognitive performance upon completion of a course of ECT. No statistically difference was found in terms of remission and response rates and serious adverse events.

One previous study performed a pairwise meta-analysis on ketamine versus propofol versus ketamine plus propofol using three RCTs,^60^ and concluded that ketamine alone and the combination of ketamine and propofol had greater efficacy in the treatment of depressive symptoms. However, this study only considered a single ECT session and excluded studies with two or more ECT sessions. Our study extends the findings of this previous study, allowing multiple comparisons across other anesthetic agents and multiple ECT sessions.

We did not find any statistically significant difference in cognitive performance in our main analysis (i.e., full network meta-analysis). This finding is in line with one systematic review, which concluded that, when compared to placebo, ketamine does not mitigate cognitive side effects associated with ECT.^61^ In our sensitivity analysis comparing ketamine versus propofol versus ketamine plus propofol, however, ketamine treated individuals demonstrate worse cognitive performance than propofol, a reference group, albeit the effect size was small.

When reviewing articles qualitatively, only 4 studies^40,43,44,51^ reported long-term (i.e., 3 or more months) follow-up outcomes. These studies had a follow-up period of 3 to 6 months and reported no significant differences in terms of depressive symptoms and cognitive performance. One study also reported a relapse rate of depression during the 6-month follow-up, and no significant difference was found between ketamine and propofol treatment arms.^43^

The current study has several clinical implications. First, ketamine has been used as a general anesthetic agent since 1960s,^17^ and RCTs of ketamine-assisted ECT have been introduced since early 2010s. The current network meta-analysis demonstrates that ketamine-assisted ECT is not only tolerable, but may even be efficacious in improving depressive symptoms in some patients with MDE. However, because ketamine may be associated with additive cognitive side effects (e.g., a higher incidence of confusion) and/or delayed time to recovery when compared to other anesthetic agents,^62^ the selection of anesthetic should be carefully assessed prior to the use of ECT. Second, for some patients, ketamine infusion alone without ECT may also be an option because ketamine itself has shown to have rapid and robust antidepressant effects in patients with MDD,^63,64^ although ECT may be more efficacious than ketamine in improving depressive symptoms.^14^ Such clinical decisions should include a careful informed consent process taking into consideration of known risks and potential benefits in addition to provider- and patient-level barriers and/or preferences.

This study has several limitations. First, unlike a standard, pairwise meta-analysis comparing only two interventions at a time, a network meta-analysis (or multiple treatments meta-analysis) has its own strengths and weaknesses.^65^ For example, the validity of the results of a NMA depends on the soundness of several assumptions: similarity (across trial designs, study settings, etc.), transitivity, consistency, and coherence. Our network structure was not connected across all intervention types, and some of our nodes were considered a star or complex network. While we did not violate methodological assumptions, due to the small number of trials and our reliance on aggregate (rather than patient-level) data, we cannot rule out the possibility that confounding factors may potentially threaten our analytical interpretations.

Second, our findings are limited in that we were unable to assess efficacy and safety of ketamine versus other agents among specific subgroups (e.g., older patients, and those with psychotic features or with psychiatric multi-morbidities) due to insufficient data to form a consistent network. Third, we were not able to assess other potentially important outcomes (e.g., cardiovascular events) due to insufficient data or differences in the management after the ECT intervention. These limitations should be considered when interpreting our findings.

## Conclusions

In conclusion, this systematic review and network meta-analysis suggests that ketamine-assisted ECT, when compared to other anesthetic agents, may be efficacious in improving depressive symptoms in the acute phase treatment of a MDE, but cognitive performance may be an important counter-balancing risk factor. ECT practitioners should consider different anesthetic agents based on clinical efficacy and safety, while taking into account specific patient clinical profiles and preferences to improve effectiveness and safety of ECT for major depression.

## Supplementary Material

Supplement 1

## Figures and Tables

**Table 1:** Characteristics of the randomized controlled trials (RCTs) included (N = 22)

Study (Year^a)^)	Country	Study design	Condition	Ketamine [dose] (n; mean age; % male sex)	Control [dose] (n; mean age; % male sex)	ECT characteristic	Duration^b)^	Key finding
Abdallah (2012)	USA	RCT	Unipolar or bipolar depression; No history of psychosis	thiopental + ketamine [3.5mg/kg + 0.5mg/kg] (8; 47.8; 63)	thiopental [3.5mg/kg] (8; 46.5; 50)	3 times/week for 6 sessions; Unilaterally or bilaterally via a SpECTrum 5000 Q	2 weeks	Ketamine did not improve the antidepressant effect of ECT.
Alizadeh (2015)	Iran	Double-blind RCT	Major depressive disorder	propofol + ketamine [1 mg/kg + 0.3 mg/kg] (22; 34.3; 27)	propofol [1 mg/kg] (22; 35.1; 35)	3 times/week for 6 sessions; Bilaterally	2 weeks	No significant difference in depression severity between groups; However, cognitive performance recovery time was lower in the ketamine group.
Altinay (2019) *	USA	Double-blind RCT	Unipolar or bipolar depression; No history of psychosis	ketamine [0.5 mg/kg] (7; 39; 15)	midazolam [0.045 mg/kg] (5; 38; 20)	2–3 times/week; Bilaterally	3 weeks	No significant difference in depression severity between groups; However, the ketamine group showed early remission and maintained euthymia.
Anderson (2017)	UK	Double-blind RCT	Unipolar or bipolar depression	ketamine [0.5 mg/kg] (33; 52.5; 33)	propofol (or thiopental) [n/a] (37; 56.4; 40)	2 times/week; Unilaterally or bilaterally using Thymatron IV or Mecta Spectrum 5000	4 weeks	No evidence to support the use of adjunctive low-dose ketamine in routine ECT treatment.
Brunelin (2020)	France	Double-blind RCT	Unipolar or bipolar depression	propofol + ketamine [n/a + 0.5 mg/kg] (11; 57.3; 64)	propofol + placebo [n/a] (16; 59.6; 56)	2 times/week; Unilaterally or bilaterally via a SpECTrum 5000Q	4 weeks	No evidence to support the use of the combination of ketamine and propofol as an anesthetic agent for ECT.
Carspecken (2018)	USA	Double-blind RCT	Unipolar or bipolar depression	ketamine [1–2 mg/kg] (23; 47; 89)	Methohexital [1–2 mg/kg] (27; 47; 89)	3 times/week; Unilaterally or bilaterally	2–4 weeks	Ketamine does not significantly improve depression when compared with methohexital.
Chen (2020) *	China	Double-blind RCT	Major depressive disorder; No history of psychosis	propofol + ketamine [1.5 mg/kg + 0.3 mg/kg] (63; 40.9; 33)	propofol [1.5 mg/kg] (64; 37.4; 36)	3 times/week for 12 sessions; Bilaterally	4 weeks	No significant differences were found in the overall response, remission and relapse rates between the groups.
Dong (2019)	China	Double-blind RCT	Major depressive disorder; No history of psychosis	propofol + ketamine [1–1.5 mg/kg + 0.3 mg/kg] (43; 36.8; 42)	propofol [1–1.5 mg/kg] (45; 35.7; 49)	3 times/week for 6–15 sessions; Bilaterally using Thymatron System	2–5 weeks	Ketamine-assisted ECT achieved a higher remission rate.
Fernie (2017)	UK	Double-blind RCT	Unipolar or bipolar depression	ketamine [up to 2 mg/kg] (16; 51.8; 56)	propofol [up to 2.5 mg/kg] (17;49.9; 53)	2 times/week; Bilaterally using a brief Pulse constant current apparatus (Thymatron DGx)	3–4 weeks	Ketamine as an anesthetic does not enhance the efficacy of ECT.
Gamble (2018)	Canada	Double-blind RCT	Major depressive disorder	ketamine [0.75 mg/kg] (12; 42; 50)	propofol [1 mg/kg] (12; 46; 50)	8 sessions; Unilaterally or bilaterally	3–4 weeks	ketamine-based anesthesia, compared withpropofol-based anesthesia, provided response and remission after fewer ECT sessions.
Jarventausta (2013)	Finland	RCT	Major depressive disorder	propofol + ketamine [n/a + 0.4 mg/kg] (16; 48.8; 50)	propofol [n/a] (16; 53.7; 31)	6 sessions; Unilaterally or bilaterally	2–3 weeks	Adjuvant dose of S-ketamine with propofol may not increase the effects of ECT in patients with treatment-resistant depression.
Loo (2012)	Australia	Double-blind RCT	Unipolar or bipolar depression	ketamine [0.5 mg/kg] (22; 45.2; 50)	placebo [n/a] (24; 41.4; 29)	3 times/week for 6 sessions; Unilaterally using a Mecta Spectrum 5000	2 weeks	Ketamine did not decrease cognitive impairment, but was safe and slightly improved efficacy in the first week of treatment and at one-week follow up.
Okamoto (2010) *	Japan	Double-blind RCT	Major depressive disorder	ketamine [0.75 mg/kg] (11; 59.3; 45)	propofol [1 mg/kg] (20; 55.1; 50)	2 times/week for 8 sessions; n/a	4 weeks	It is possible to improve symptoms of depression earlier by using ketamine anesthesia.
Rasmussen (2014)	USA	RCT	Unipolar or bipolar depression	ketamine [1 mg/kg] (21; 47; 24)	methohexital [1 mg/kg] (17; 48.6; 53)	6 sessions; Unilaterally or bilaterally	2–3 weeks	There were no significant differences in depression or cognitive outcomes between the two drugs.
Ray-Griffith (2017)	USA	RCT	Unipolar or bipolar depression	ketamine [1 mg/kg] (8; 43.6; 25)	methohexital [1 mg/kg] (8; 38.1; 13)	3 times/week and up to 6 sessions; Unilaterally	2 weeks	No statistical difference was found between the ketamine and methohexital groups for an improvement in depressive symptoms.
Salehi (2015)	Iran	Double-blind RCT	Major depressive disorder	ketamine [0.8 mg/kg] (80; n/a; 45)	thiopental [1–1.5 mg/kg] (80; n/a; 43)	3 times/week for 8 sessions; n/a	3–4 weeks	Ketamine is an appropriate option for anesthesia with ECT in patients with drug-resistant major depression.
Wang (2012) *	China	RCT	Major depressive disorder	ketamine [0.8 mg/kg] (12; 56.2; 50) propofol + ketamine [1.5 mg/kg + 0.8 mg/kg] (16; 58.6; 58)	propofol [1.5 mg/kg] (12; 53.8; 42)	Single treatment; Bilaterally	1 day	Propofol combined with ketamine may be the first-choice anesthesia in patients with depressive disorder undergoing ECT.
Woolsey (2022)	Canada	Double-blind RCT	Major depressive disorder	ketamine [0.2–0.5 mg/kg] (16; 36.9; 31.3)	propofol [0.2–0.5 mg/kg] (15; 45.0; 26.7)	3 times/week up to 12 sessions; Unilaterally or bilaterally via a Mecta SpECTrum 5000Q	4–5 weeks	Ketamine does not improve psychiatric outcomes in ECT.
Yoosefi (2014) *	Iran	Double-blind RCT	Major depressive disorder	ketamine [1–2 mg/kg] (15; 40.9; 53)	thiopental [2–3 mg/kg] (14; 47; 57)	3 times/week and 6 sessions; Bilaterally	2–3 weeks	Ketamine administration during ECT is well tolerated and patients may experience earlier improvement in depressive symptoms, longer seizure duration, and better cognitive performance when compared with thiopental.
Zhang (2018)	China	Double-blind RCT	Unipolar or bipolar depression	propofol + ketamine [0.5 mg/kg + 0.5 mg/kg] (16; 48.8; 50)	propofol [0.5 mg/kg] (16; 53.7; 31)	3 times/week for 6 sessions; Bilaterally using Thymatron System	2 weeks	Ketamine plus propofol anesthesia in the ECT treatment of MDD and BP was not superior on any measure to propofol alone.
Zhong (2016)	China	RCT	Unipolar or bipolar depression	ketamine [0.8 mg/kg] (30; 32.1; 47) propofol + ketamine [0.5 mg/kg + 0.5 mg/kg] (30; 30.4; 40)	propofol [0.8 mg/kg] (30; 29.2; 33)	3 times/week for 8 sessions; Bilaterally using Thymatron System	3 weeks	ECT with ketamine anesthesia might be an optimized therapy for patients with treatment-resistant depression.
Zou (2021)	China	Double-blind RCT	Major depressive disorder	propofol + ketamine [1.5 mg/kg + 0.3 mg/kg] (67; 65.8; 36)	propofol [1.5 mg/kg] (70; 65.6; 33)	3 times/week for 6 sessions; n/a; used Thymatron DGx	2 weeks	Low-dose ketamine is safe as an adjunct anesthetic for elderly patients subjected to ECT. It has a protective effect on cognitive function and may accelerate the antidepressant effects of ECT.

Note:

a) indicates publication year

b) denotes a period for completing a series of treatment sessions.

ECT=Electroconvulsive therapy.

* This study was not included in the meta-analysis due to insufficient data.
