# Supplementary material for: Efficacy and Safety of Ketamine-assisted Electroconvulsive Therapy in Major Depressive Episode: A Systematic Review and Network Meta-Analysis
Source: Res Sq. 2023 Aug 7:rs.3.rs-3182771. Preprint. [Version 1] doi: 10.21203/rs.3.rs-3182771/v1 (PMC10441463; doi:10.21203/rs.3.rs-3182771/v1)
Supplement: Supplement 1 [file NIHPPrs3182771v1-supplement-1.pdf]

Supplementary Files

This is a list of supplementary files associated with this preprint. Click to download.

- MolecularPsychKETassistedECTSupplement.docx
